# The effects of different implant crown restorative materials on the detection of proximal caries of adjacent teeth in cone-beam computed tomography (CBCT) scans using various machines, metal artifact-reduction (MAR) algorithm, and exposure protocols

**DOI:** 10.4317/jced.63883

**Published:** 2026-03-30

**Authors:** Mina Morcos, Ben Bartlett, Rujuta Katkar, Taraneh Maghsoodi, Hassem Geha, Nikolaos Shinas

**Affiliations:** 1 BDS, Resident in Oral and Maxillofacial Radiology Master’s program, Division of Oral and Maxillofacial Radiology, Department of Comprehensive Dentistry. University of Texas, Health Science Center at San Antonio, 7703 Floyd Curl Dr., San Antonio, TX, 7822, USA; 2 DMD, Resident in Oral and Maxillofacial Radiology Master’s program, Division of Oral and Maxillofacial Radiology, Department of Comprehensive Dentistry. University of Texas, Health Science Center at San Antonio, 7703 Floyd Curl Dr., San Antonio, TX, 7822, USA; 3 BDS, MDS, MS, Associate Professor, Division of Oral and Maxillofacial Radiology, Department of Comprehensive Dentistry. University of Texas, Health Science Center at San Antonio, 7703 Floyd Curl Dr., San Antonio, TX, 7822, USA; 4 DDS, MS, Clinical Assistant Professor, Division of Oral and Maxillofacial Radiology, Department of Comprehensive Dentistry. University of Texas, Health Science Center at San Antonio, 7703 Floyd Curl Dr., San Antonio, TX, 7822, USA; 5 DDS, MDS, Professor and Director, Division of Oral and Maxillofacial Radiology, Department of Comprehensive Dentistry. University of Texas, Health Science Center at San Antonio, 7703 Floyd Curl Dr., San Antonio, TX, 7822, USA; 6 DDS, MS, Clinical Assistant Professor, Department of Oral and Maxillofacial Radiology. Boston University, Henry M. Goldman School of Dental Medicine, 635 Albany St, Boston, MA, 02118, USA

## Abstract

**Background:**

This study aimed to investigate how artifacts from different restorative materials affect the detection of approximal caries in CBCT scans, using various machines, artifact-reduction (AR) algorithms, and exposure protocols.

**Material and Methods:**

Ten permanent teeth (5 molars, 5 premolars) were selected and categorized into 3 intact and 7 with artificial carious lesions. The teeth were arranged in a human mandible with an ingot of material in-between and in the following approximal surfaces configurations: intact-ingot-intact, caries-ingot-caries, and intact-ingot-caries/caries-ingot-intact. Three different ingot materials were used (3Y and 5Y Zirconia, and E-Max) and the setup was scanned using two CBCT systems (Planmeca Mid and Prexion) with varying exposure protocols, with and without AR algorithm, thus resulting in 90 scans. Three observers of different experience levels evaluated the images and P and Kappa values were calculated.

**Results:**

Kappa and P values showed a significant positive association with the AR option, and a negative association without AR for zirconia 5Y. Zirconia 3Y had the highest positive association across all observers using both low and standard exposure settings on the Prexion®. The inter-observer agreement was insignificant, while intra-observer agreement was highly significant.

**Conclusions:**

The AR option improves the accuracy of approximal caries detection in CBCT scans for materials with higher yttria concentration but has no significant effect on lower yttria materials. The Prexion® scanner outperformed the Planmeca ProMax 3D Mid® in caries detection without AR.

## Introduction

Caries is one of the most prevalent chronic diseases worldwide affecting a significant portion of the population, with prevalence rates varying by age group, geographic region, and socioeconomic factors. Its prevalence can range from 60% to 90%, while in adults, it is estimated to affect nearly 100% of individuals at some point in their lives (1). Many diagnostic tools have been developed over the years to assist diagnosis, exactly due to the high prevalence of the disease. However, radiographs are commonly used as the primary approach (2). Radiographic visualization includes the use of both intraoral and extraoral techniques. Intraoral techniques like bitewing radiographs, which have been the golden standard of the industry, are highly effective for detecting interproximal caries, especially in the posterior teeth and at early stages with an average accuracy range between 60% and 85% (3). Extraoral techniques most commonly involve the use of panoramic radiographs, a simpler tool that can still provide valuable information in certain cases. They offer a broad overview of the entire dentition, jaws, and surrounding structures, making them useful for identifying gross carious lesions, especially in patients with extensive decay or when other radiographic methods are not feasible. However, their lower resolution compared to bitewings translates to reduced diagnostic accuracy (3). Another extraoral technique, that in the last 25 years has gained significant popularity in everyday clinical practice, is Cone Beam Computed Tomography (CBCT). A specialized imaging technique that uses a cone-shaped X-ray beam, CBCT can provide detailed three-dimensional images of the teeth, jaws, and surrounding structures. It operates by a rotating X-ray source and detector that move around the patient capturing multiple two-dimensional images (Basis Images) from different angles. These images are then reconstructed by specialized software to create a volumetric 3D representation of the area of interest (4). Traditionally, CBCT has found limited use in caries detection in clinical practice due to limiting factors (5), one of those being metal artefact from adjacent restorations. Metal artefact are distortions or streaks that appear on radiographic images produced by metal objects, such as dental restorations, implants, or surgical hardware. They are the product of metal interactions with X-rays, causing phenomena like beam hardening, scatter, or photon starvation. As a result, the image may show bright streaks, dark bands, or areas of reduced clarity, which can limit diagnostic details (5 , 6). Typically, metal artefact in CBCT is measured with quantitative and qualitative methods. Quantitative methods include: 1. Artefact Index (AI): This involves calculating the difference in pixel intensity between the artefact-affected area and the surrounding unaffected regions. 2. Signal-to-Noise Ratio (SNR): Evaluates the clarity of the image by comparing the signal strength to the background noise. 3. Contrast-to-Noise Ratio (CNR): Assesses the visibility of structures by comparing the contrast between the artefact and the anatomical features. Qualitative methods involve visual evaluation of the extent and impact of the artefacts on diagnostic images. This is often done using standardized scoring systems or subjective grading. Finally, advanced techniques have been developed utilizing software to simulate and analyze artefacts, providing detailed metrics on their size, intensity, and distribution (6). This study aims to explore the effect of artefact produced by the three most commonly used implant crown materials (lithium disilicate, Zirconia 3Y and 5Y) on the detection of proximal caries using two CBCT systems, with and without the use of Metal Artefact Reduction (MAR) algorithm and different exposure protocols.

## Material and Methods

For the following study, IRB exemption was obtained for the use of extracted human teeth and human mandible by UT Health, San Antonio IRB office, which determined that the proposed activity (STUDY00001157) is not regulated research involving human subjects as defined by DHHS regulations at 45 CFR 46 and FDA regulations at 21 CFR 56. Ten sound teeth that were extracted for orthodontic or periodontal reasons (five permanent molars and five permanent premolars) with no clinical or radiographic caries, hypoplastic pits or restorations were selected with a total of ten approximal surfaces. Seven artificial cavities were created on seven teeth using a high-speed hand piece and 245-carbide bur by a general dentist with three years of experience. Cavity extensions ranged from outer one third of enamel (E1), middle one-third of the enamel (E2), and middle one-third of the dentin (D2) (7). Simulated caries Telio ® Inlay/Onlay, (Ivoclar®) was placed in the prepared cavities. For the purpose of replicating metal artefact from adjacent restorations, ingots of materials were created. The selection of materials was based on popularity, and included lithium disilicate glass-ceramic (LS) (IPS e.max, Ivoclar, Liechtenstein, Germany) commonly known as Emax, Zirconia 3Y, and Zirconia 5Y via NexxZr T. The ingots were fabricated at a private dental laboratory (Art and Technology Dental Studio, San Antonio, TX) to replicate best the dimensions of the LS ingot (12 millimeters in height and 9.5 millimeters in diameter). The zirconia ingots measured 12 millimeters in height and 9 millimeters in diameter. Teeth were numbered T1-T10 and five randomized orientations were created allowing for all different scenarios: intact-ingot-intact, intact-ingot-cavity, cavity-ingot-intact, and cavity-ingot-cavity, resulting in the following combinations: 1. T1 - ingot - T3 2. T6 - ingot - T2 3. T8 - ingot - T4 4. T7 - ingot - T9 5. T5 - ingot - T10 Teeth were then fixed in their positions on the mandible based on the pre-selected orientation using utility wax for stabilization with the molars in site #18, premolars in #20 and the ingot in #19. Proximal contacts were assessed using dental floss. For the purposes of the study CBCT scanners Planmeca® ProMax 3D mid-2015 (Planmeca, Helsinki, Finland) and PreXion® 3D Excelsior (PreXion Inc., San Mateo, USA) were used, located at the Center for Oral Health Care and Research in San Antonio, Texas. A plastic transparent cylindrical jar was used as a phantom to place the mandible in, marked at both condylar head sites and anterior midline to ensure reproducibility. The phantom was filled with a medium of isotonic saline (0.9% NaCl) to replicate soft tissues. A power analysis was performed and determined that 87.88 total scans were required. Six different exposure settings were selected, low and standard with and without Metal Artefact Reduction (MAR) option on Planmeca®, and low and standard on PreXion. Each acquisition on the Planmeca® used the mandibular 8 cm x 5 cm FOV and the medium adult size. The scanning parameters for low dose and normal were set to their default settings, with the low dose settings being 400 mm voxel size, 90 kVp, 5.6 mA, 6-second scan time, and a CTDI (Computed Tomography Dose Index) 2.8 mGy and the normal dose settings being 200 mm voxel size, 90 kVp, 8 mA, 12-second scan time, and CTDI 8.0 mGy. Each acquisition on the PreXion used the mandibular 10 cm x 5 cm FOV and the medium adult size. The scanning parameters for rapid and standard were set to their default settings which could not be altered. The rapid dose settings were 200 µm voxel size, 110 kVp, 2.4 mA, 2.3-second scan time, and CTDI 1.3 mGy and the standard dose settings were 200 µm voxel size, 110 kVp, 2.4 mA, 7.7-second scan time, and CTDI 4.1 mGy. Each configuration underwent five acquisitions at each parameter listed above using the pre-selected teeth orientations (T1-ingot-T3, T6-ingot-T2, T8-ingot-T4, T7-ingot-T9, T5-ingot-T10). For all Planmeca® scans, the proprietary motion artifact reduction algorithm called CALM (Corrective Adaptive Logic for Motion) was turned off during acquisition and the MAR algorithm was applied post-scan at "Mid" level. The rationale for selecting "Mid" after the initial acquisition was that it is the manufacturer's default setting, therefore the most commonly used by clinicians, and to avoid altering the raw data of the scan (8). For both machines, no post-scan changes were made to contrast, brightness or any other image parameters. The exported slice thickness was selected to reflect the voxel size of the parameter used (i.e., for a low dose scan with 400 µm voxel size, the chosen slice thickness was 0.400 mm). On the PreXion, the "Use original pitch" option was selected to reflect the actual voxel size. A total of 90 scans were executed by a third-year maxillofacial radiology resident and the scans were randomized and examined by three Oral &amp; Maxillofacial radiologists with different level of experience. Observer 1 with over 20 years of experience, observer 2 with over six years of experience, and observer 3 a first-year oral maxillofacial radiology resident. The proximal mesial and distal surfaces of the two teeth located mesial and distal to the ingot were evaluated. For each proximal surface, the observers were asked to state whether they could identify caries using a 5-step confidence scale as follows: 1, caries-free; 2, possible caries-free; 3, not sure; 4, possible caries; 5, caries. The observations and scoring were performed using one threshold during one session. All observers assessed the images in a dimmed room on the same computer, with a Dell UltraSharp U2312HM 23" IPS LED LCD (Dell®, Round Rock, Texas, USA) monitor and at a distance approximately 60 cm from the viewer. Invivo 7 software (Osteoid Inc., Santa Clara, California, USA) was used to display images for evaluation. The observers were able to modify the brightness, contrast, and gamma parameters of each slice according to their personal preference. For evaluation purposes, each observer could choose any or all of the images provided and focus on the preferred images to visualize any possible caries on the proximal surfaces. The observers had access to the entire volume of scans, allowing them to scroll freely through the slices in the three axes (axial, coronal, and sagittal) and/or any oblique direction for better assessment. Thirty different random scans were re-selected for each observer and evaluated two weeks later for the intra-observer reliability. Inter-observer and intra-observer agreements were calculated using a weighted interclass correlation coefficient and Cronbach's Alpha. Cohen's Kappa value for measurement of agreement between original and observers. A P value of &lt; 0.05 was considered statistically significant and p &lt; 0.001 as highly significant. Kappa value was interpreted as follows 0; no agreement, 0.10-0.20; Slight agreement, 0.21-0.40; fair agreement, 0.41-0.60; moderate agreement, 0.61-0.80; substantial agreement, 0.81-0.99; near perfect agreement, 1; perfect agreement. All analysis was performed using SPSS 23.0 version.

## Results

Results were divided into four subclasses: overall performance of the three observers, overall performance of both machines, material performance on each scanner, and lastly the highest and lowest image quality produced by all different setting using both scanners. For the overall performance of the three observers no statistically significant results were noted concluding that experience did not aid in better detections in this study (Table 1).

Table 1

Both the PreXion® 3D Excelsior® and the Planmeca® ProMax 3D Mid 2015® revealed statistically insignificant associations, with the PreXion® 3D Excelsior® showing slightly higher values (Table 2).

Table 2

**Figure 1:**
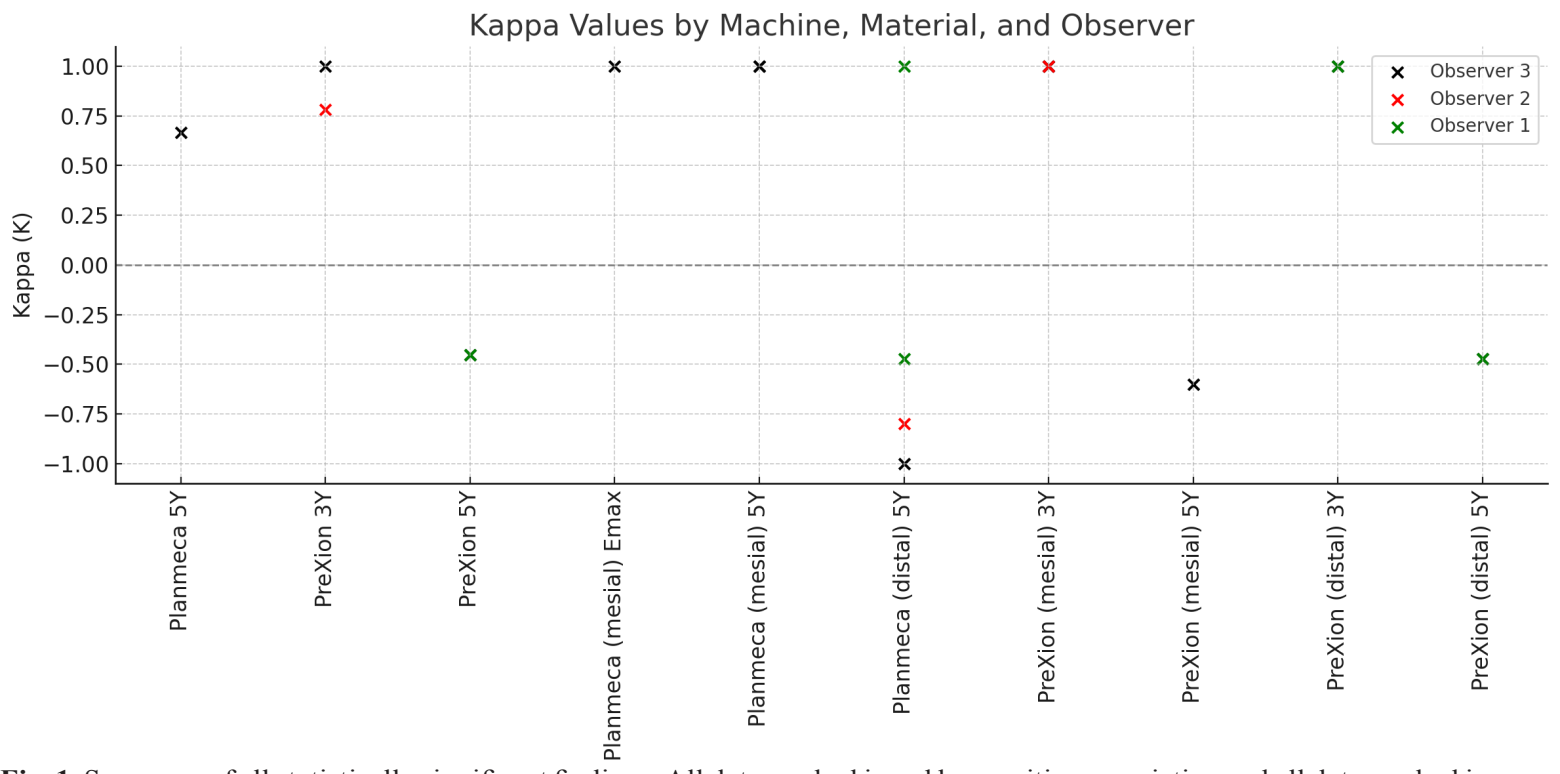
Summary of all statistically significant findings. All data marked in red has positive association and all data marked in green has negative association.

In regard to machine performance using different materials, the Planmeca® scanner revealed statistically significant results with positive correlation for 5Y and Observer 3 (K 0.667, p 0.021) (Tables 3-7, Fig. 1).

Table 3

Table 4

Table 5

Table 6

Table 7

1

As for the PreXion scanner, it revealed statistically significant results with positive association for 3Y and Observers 2 and 3 (K 0.783, p 0.011 and K 1.000, p 0.014 respectively). Observer 1 had barely statistically insignificant results showing a positive association with 3Y (K 0.571, p 0.058). On the other hand, statistically significant results with negative association for 5Y and Observers 1 and 3 (K -0.452, p 0.016 and K -0.452, p 0.016, respectively) were noted and Observer 2 had barely statistically insignificant results showing a negative association with 5Y (K -0.429, p 0.053) (Tables 3-7, Fig. 1), thus emphasizing the benefits of using the available MAR algorithm on Planmeca® with higher scatter producing restorative materials such as the zirconia 5Y. Additionally the higher kVp settings on the PreXion® scanner effectively reduced scatter when imaging the Emax and zirconia 3Y without the use of MAR. As for the exposure settings, 5Y on the Planmeca® had the highest positive association using the MAR option and ranked 1st on the Low+MAR with a statistically significant positive association (K 1.000, p 0.046 and K 1.000, p 0.083) by Observers 1 and 3. Standard+MAR came in 2nd with a statistically significant positive association (K 1.000, p 0.046) by Observer 3, Emax using the Standard setting produced statistically significant positive association (K 1.000, p 0.083) by Observer 3, the 3Y using the Standard settings was the 2nd lowest of all settings with a barely not statistically significant results with a negative association (K -0.800, p 0.083) by observer 3, and finally 5Y using the Standard setting was the lowest of all with a statistically significant negative association (K -0.471, p 0.025; K -0.800, p 0.083; K -1.000, p 0.157 respectively) by Observers 1, 2 and 3 (Tables 4-7, Fig. 1). However, different results were noted on the PreXion® scanner. The 3Y ranked 1st using both the Low and Standard setting showing statistically significant results with a positive association for Observers 2 and 3 (K 1.000, p 0.025 and K 1.000, 0.083 respectively) by the Low setting, and statistically significant results with positive association for Observers 1 and 3 (K 1.000, p 0.083 and K 1.000, p 0.046 respectively) by the Standard setting. Emax showed no statistically significant results on all settings, while 5Y again ranked lowest using the Standard setting producing statistically significant results with negative association for Observers 1 and 3 (K -0.471, p 0.025; K -0.471, p 0.025 respectively), and statistically significant results with a negative association for Observer 3 (K -0.600, p 0.046) using the Low setting (Table 6, Fig. 1). For Intra-observer reliability, all p values were highly statistically significant &lt;0.001 with Observer 1 having the highest ICC and the narrowest confidence interval suggesting excellent reliability. Observer 2 also demonstrated strong agreement but with a slightly wider confidence interval hinting to the presence of some variations, and finally Observer 3 had the lowest ICC and much wider confidence interval indicating a relatively lower but still good reliability (Table 6, Fig. 1).

## Discussion

Caries is one of the most common dental diseases affecting nearly everyone at some point in life. Early detection aids in the prognosis and help reserving the teeth and their vitality (9). Different diagnostic tools exist, but radiographs remain the best method, with bitewing radiographs offering the highest accuracy for detecting early interproximal caries (3 , 9). The findings of this study highlight major obstacles in caries detection, particularly in the presence of metal artifacts produced by different metallic and non-metallic restorative materials affecting CBCT imaging. These artifacts-primarily resulting from beam hardening, scatter, and photon starvation, complicate clinical and radiographic assessments, especially when evaluating proximal caries adjacent to restorative materials. The effectiveness of metal artifact reduction algorithms, alongside optimized exposure settings using different scanners to lessen these limitations was explored in our study. The diagnostic performance of two CBCT systems, Planmeca® ProMax 3D Mid 2015 and PreXion 3D Excelsior®, showed variability in sensitivity and specificity depending on imaging parameters and the restorative materials analyzed. Proximal surfaces adjacent to zirconia ingots exhibited significant differences in detection rates, which could be attributed to their Yttria content. With the Planmeca® system, zirconia 5Y demonstrated improved caries detection when MAR was applied under standard and low settings, which could be attributed to the higher cubic content phase with larger particles and higher translucency. A higher Yttria content allows for more radiation scatter and increased adjacent opacity validating the utility of artifact reduction tools in refining diagnostic accuracy in high metallic content restorative materials. Our results are in alliance with a study done by Cebe et al. confirming the benefits of using AR option with metallic content materials such as amalgam (10 , 11). In contrast, under standard exposure settings without MAR, zirconia 5Y was associated with a considerable decrease in detection performance (K=-0.471, P=0.025), further emphasizing the necessity of algorithm integration in CBCT workflows. Similar results were observed with the PreXion® 3D Excelsior® system, where zirconia 3Y demonstrated enhanced caries detection across both low and standard exposure settings (K=1.000, P=0.025 for low; K=1.000, P=0.046 for standard) a result of its predominantly tetragonal phase composition and smaller particles, exhibiting reduced adjacent scatter (11). These results confirm how both exposure settings and material composition influence diagnostic outcomes. The observed decline in performance with zirconia 5Y (K=-0.471, P=0.025) bolsters the need for specialized imaging protocols when assessing lesions adjacent to high-density restorations. Based on the results of our study the following protocols are suggested for optimal diagnostic quality: 1. For Planmeca®: a. Emax: Standard 200m. b. 3Y: No statistical significance recorded between protocols. c. 5Y: Low 400 µm + MAR. 2. For PreXion®: a. Emax: Standard 200 µm. b. 3Y: Rapid 200 µm. c. 5Y: Rapid 200 µm. Observers showed strong agreement on key imaging parameters, which reinforces the reliability of CBCT systems, especially when paired with the right exposure settings and processing techniques. Keeping protocols consistent helps reduce variations and improve accuracy in clinical and radiographic diagnoses. Our study also emphasizes that automatically applying MAR when a patient is known to have restorative materials could have an adverse effect. This was also shown in other studies where they advised on the application of MAR depending on the diagnostic objectives (10 , 12 , 13). Cebe et al. measured the sensitivity and specificity of caries detection adjacent to restorative materials using CBCT with and without AR. They revealed that amalgam restorations caused the most artifacts, leading to high false positives in caries detection. AR increased specificity but decreased sensitivity, hinting that while AR algorithms improve visualization, they may introduce slight resolution loss suggesting a trade-off between artifact reduction and image detail (10). This was also confirmed by Bechara et al. while testing the effectiveness of AR on the detection of root fractures in the presence of gutta-percha, revealing lower accuracy when using AR on two different scanners. They found that the accuracy was highest when the MAR was turned off (12). Parrone et al. evaluated root fracture detection in endodontically treated teeth by varying CBCT parameters utilizing an optimization filter. They concluded that even though newer CBCT features exist, they may not always directly aid in detecting fractures (13). Contrary to the initial hypothesis that Emax was expected to have a significantly higher positive association for all observers, results for the three observers, on both machines and under all exposure settings revealed a non-significant association. This could be attributed to the better image quality with less artifacts for the Emax compared to the other materials, resulting in a higher number of false positive due to diagnostic bias. Hence, small anatomical variations or minor defects could have been over-diagnosed as caries. In addition, the similar brightness of Emax and the adjacent enamel can lead to Mach band effect, thus exaggerating contrast and over-diagnosis. Limitations and future research Our study provides a foundational perspective on how restorative materials, imaging parameters, and artifact reduction strategies interact in CBCT-based caries detection; however, the study's in vitro design and lack of inter-operator variability limit direct generalization of the results in real-life scenarios. Firstly, the in vitro design does not fully replicate clinical conditions. Multiple parameters, such as patient motion, anatomical variability and soft-tissue attenuation play a crucial role in caries detection on CBCT. Furthermore, the presence of metal artefact from adjacent restorations on real-life patients further limits the application of the results on clinical scenarios. Ultimately, in vitro studies like this one may be over-estimating the diagnostic capability of the modality examined. Finally, the absence of inter-operator variability could influence measurements or interpretations. A single operator ensured data reproducibility, though this limits interoperability-a recognized flaw. Future studies should focus on refining MAR algorithms to further reduce metal artifacts, particularly in cases involving high-density restorative materials. Additionally, exploring alternative imaging modalities and advanced software solutions may enhance caries detection, especially for early-stage lesions that remain challenging to visualize.

## Figures and Tables

**Table 1:** Overall results between observers with no statistically significant differences.

	Original	Observer 1			Observer 2			Observer 3		
		NC	C	Kappa stats	NC	C	Kappa stats	NC	C	Kappa stats
Mesial	NC	27	8	κ= 0.058p= 0.500	21	13	κ= 0.144p= 0.183	13	9	κ= 0.126p= 0.325
		77.1%	22.9%		61.8%	38.2%		59.1%	40.9%	
	C	36	15		22	25		16	19	
		70.6%	29.4%		46.8%	53.2%		45.7%	54.3%	
Distal	NC	9	9	κ= -0.017p= 0.854	4	11	κ= -0.075p= 0.441	4	12	κ= -0.116p= 0.309
		50.0%	50.0%		26.7%	73.3%		25.0%	75.0%	
	C	32	29		26	44		21	33	
		52.5%	47.5%		37.1%	62.9%		38.9%	61.1%	

1

**Table 2:** Comparison of the two CBCT systems used in the study. Both the PreXion® 3D Excelsior® and the Planmeca® ProMax 3D Mid 2015® revealed statistically insignificant associations, with the PreXion® 3D Excelsior® showing slightly higher values.

Machine		Original	Observer 1			Observer 2			Observer 3		
			NC	C	Kappa stats	NC	C	Kappa stats	NC	C	Kappa stats
Planmeca	Mesial	NC	18	6	κ= 0.048p= 0.660	13	9	κ= 0.103p= 0.442	7	7	κ= 0.135p= 0.418
			75.0%	25.0%		59.1%	40.9%		50.0%	50.0%	
		C	23	10		15	16		8	14	
			69.7%	30.3%		48.4%	51.6%		36.4%	63.6%	
	Distal	NC	6	6	κ= 0.01p= 0.879	1	8	κ= -0.174p= 0.160	1	9	κ= -0.188p= 0.213
			50.0%	50.0%		11.1%	88.9%		10.0%	90.0%	
		C	19	21		16	30		10	24	
			47.5%	52.5%		34.8%	65.2%		29.4%	70.6%	
PreXion	Mesial	NC	9	2	κ= 0.080p= 0.558	8	4	κ= 0.179p= 0.229	6	2	κ= 0.118p= 0.525
			81.8%	18.2%		66.7%	33.3%		75.0%	25.0%	
		C	13	5		7	9		8	5	
			72.2%	27.8%		43.8%	56.3%		61.5%	38.5%	
	Distal	NC	3	3	κ= -0.075p= 0.601	3	3	κ= 0.058p= 0.713	3	3	κ= -0.034p= 0.829
			50.0%	50.0%		50.0%	50.0%		50.0%	50.0%	
		C	13	8		10	14		11	9	
			61.9%	38.1%		41.7%	58.3%		55.0%	45.0%	

2

**Table 3:** Comparison of CBCT systems and different materials. In regard to machine performance using different materials, the Planmeca® scanner revealed statistically significant results with positive correlation for 5Y and Observer 3 (K 0.667, p 0.021).

Machine		Material	Original	Observer 1			Observer 2			Observer 3		
				NC	C	Kappa stats	NC	C	Kappa stats	NC	C	Kappa stats
Planmeca	Mesial	Emax	NC	6	2	κ= 0.191p= 0.361	3	5	κ= 0.043p= 0.848	1	3	κ= 0.000p= 1.000
				75.0%	25.0%		37.5%	62.5%		25.0%	75.0%	
			C	6	5		4	8		2	6	
				54.5%	45.5%		33.3%	66.7%		25.0%	75.0%	
		3Y	NC	5	3	κ= -0.256p= 0.163	5	3	κ= -0.041p= 0.858	1	3	κ= -0.333p= 0.221
				62.5%	37.5%		62.5%	37.5%		25.0%	75.0%	
			C	9	1		6	3		5	3	
				90.0%	10.0%		66.7%	33.3%		62.5%	37.5%	
		5Y	NC	7	1	κ= 0.182p= 0.292	5	1	κ = 0.294p = 0.182	5	1	κ= 0.667p= 0.021*
				87.5%	12.5%		83.3%	16.7%		83.3%	16.7%	
			C	8	4		5	5		1	5	
				66.7%	33.3%		50.0%	50.0%		16.7%	83.3%	
	Distal	Emax	NC	2	2	κ= -0.059p= 0.771	1	3	κ= -0.098p= 0.639	1	3	κ= 0.000p= 1.000
				50.0%	50.0%		25.0%	75.0%		25.0%	75.0%	
			C	7	5		6	10		3	9	
				58.3%	41.7%		37.5%	62.5%		25.0%	75.0%	
		3Y	NC	2	2	κ= 0.113p= 0.605	0	3	κ= -0.246p= 0.259	0	4	κ= -0.364p= 0.159
				50.0%	50.0%		.0%	100.0%		.0%	100.0%	
			C	5	9		5	11		4	7	
				35.7%	64.3%		31.3%	68.8%		36.4%	63.6%	
		5Y	NC	2	2	κ= 0.000p= 1.000	0	2	κ= -0.217p= 0.308	0	2	κ= -0.226p= 0.400
				50.0%	50.0%		.0%	100.0%		.0%	100.0%	
			C	7	7		5	9		3	8	
				50.0%	50.0%		35.7%	64.3%		27.3%	72.7%	
PreXion	Mesial	Emax	NC	3	1	κ= -0.071p= 0.747	2	2	κ= -0.098p= 0.764	2	1	κ= -0.111p= 0.673
				75.0%	25.0%		50.0%	50.0%		66.7%	33.3%	
			C	5	1		3	2		4	1	
				83.3%	16.7%		60.0%	40.0%		80.0%	20.0%	
		3Y	NC	3	0	κ= 0.571p= 0.058	3	1	κ= 0.783p= 0.011*	2	0	κ= 1.000p= 0.014*
				100.0%	.0%		75.0%	25.0%		100.0%	.0%	
			C	2	4		0	6		0	4	
				33.3%	66.7%		.0%	100.0%		.0%	100.0%	
		5Y	NC	3	1	κ= -0.207p= 0.197	3	1	κ= -0.047p= 0.858	2	1	κ= -0.296p= 212
				75.0%	25.0%		75.0%	25.0%		66.7%	33.3%	
			C	6	0		4	1		4	0	
				100.0%	.0%		80.0%	20.0%		100.0%	.0%	
	Distal	Emax	NC	1	1	κ= 0.091p= 0.747	1	1	κ= 0.375p= 0.236	1	1	κ= 0.182p= 0.571
				50.0%	50.0%		50.0%	50.0%		50.0%	50.0%	
			C	3	5		1	7		2	5	
				37.5%	62.5%		12.5%	87.5%		28.6%	71.4%	
		3Y	NC	2	0	κ= 0.276p= 0.290	2	0	κ= 0.400p= 0.114	2	0	κ= 0.462p= 0.147
				100.0%	.0%		100.0%	.0%		100.0%	.0%	
			C	3	2		3	5		2	3	
				60.0%	40.0%		37.5%	62.5%		40.0%	60.0%	
		5Y	NC	0	2	κ= -0.452p= 0.016	0	2	κ= -0.429p= 0.053	0	2	κ= -0.452p= 0.016
				.0%	100.0%		.0%	100.0%		.0%	100.0%	
			C	7	1		6	2		7	1	
				87.5%	12.5%		75.0%	25.0%		87.5%	12.5%	

3

**Table 4:** Planmeca® scanner with different materials and different settings for the mesial position.

Material	Setting	Original	Observer 1			Observer 2			Observer 3		
			NC	C	Kappa stats	NC	C	Kappa stats	NC	C	Kappa stats
Emax	Low	NC	1	1	κ= -0.429p= 0.171	0	2	κ= -0.667p= 0.136	0	1	κ= -0.500p= 0.248
			50.0%	50.0%		.0%	100.0%		.0%	100.0%	
		C	3	0		2	1		2	1	
			100.0%	.0%		66.7%	33.3%		66.7%	33.3%	
	Standard	NC	2	0	κ= 0.286p= 0.361	2	0	κ= 0.615p= 0.136	1	0	κ= 1.000p= 0.083
			100.0%	.0%		100.0%	.0%		100.0%	.0%	
		C	2	1		1	2		0	2	
			66.7%	33.3%		33.3%	66.7%		.0%	100.0%	
	Low + MAR	NC	1	1	κ= 0.545p= 0.171	1	1	κ= 0.167p= 0.709	-	2	NA
			50.0%	50.0%		50.0%	50.0%			100.0%	
		C	0	3		1	2		-	2	
			.0%	100.0%		33.3%	66.7%			100.0%	
	Standard + MAR	NC	2	0	κ= 0.500p= 0.248	-	2	NA	-	-	NA
			100.0%	.0%			100.0%				
		C	1	1		-	3		-	1	
			50.0%	50.0%			100.0%			100.0%	
3Y	Low	NC	1	1	κ= -0.429p= 0.171	2	-	NA	1	1	κ= 0.000p= 1.000
			50.0%	50.0%		100.0%			50.0%	50.0%	
		C	3	0		3	-		1	1	
			100.0%	.0%		100.0%			50.0%	50.0%	
	Standard	NC	1	1	κ= -0.154p= 0.709	1	1	κ= -0.154p= 0.709	0	1	κ= -0.800p= 0.083
			50.0%	50.0%		50.0%	50.0%		.0%	100.0%	
		C	2	1		2	1		2	0	
			66.7%	33.3%		66.7%	33.3%		100.0%	.0%	
	Low + MAR	NC	1	1	κ= -0.500p= 0.248	1	1	κ= 0.000p= 1.000	0	1	κ= -0.500p= 0.248
			50.0%	50.0%		50.0%	50.0%		.0%	100.0%	
		C	2	0		1	1		2	1	
			100.0%	.0%		50.0%	50.0%		66.7%	33.3%	
	Standard + MAR	NC	2	-	NA	1	1	κ= 0.400p= 0.386	-	-	NA
			100.0%			50.0%	50.0%				
		C	2	-		0	1		-	1	
			100.0%			.0%	100.0%			100.0%	
5Y	Low	NC	1	1	κ= 0.167p= 0.709	1	1	κ= 0.500p= 0.248	1	1	κ= 0.500p= 0.248
			50.0%	50.0%		50.0%	50.0%		50.0%	50.0%	
		C	1	2		0	2		0	2	
			33.3%	66.7%		.0%	100.0%		.0%	100.0%	
	Standard	NC	2	-	NA	1	-	NA	-	-	NA
			100.0%			100.0%					
		C	3	-		3	-		1	-	
			100.0%			100.0%			100.0%		
	Low + MAR	NC	2	0	κ= 0.286p= 0.361	2	0	κ= 0.500p= 0.248	2	0	κ= 1.000p= 0.083
			100.0%	.0%		100.0%	.0%		100.0%	.0%	
		C	2	1		1	1		0	1	
			66.7%	33.3%		50.0%	50.0%		.0%	100.0%	
	Standard + MAR	NC	2	0	κ= 0.286p= 0.361	1	0	κ= 0.500p= 0.248	2	0	κ= 1.000p= 0.046
			100.0%	.0%		100.0%	.0%		100.0%	.0%	
		C	2	1		1	2		0	2	
			66.7%	33.3%		33.3%	66.7%		.0%	100.0%	

4

**Table 5:** Planmeca® scanner with different materials and different settings for the distal position.

Material	Setting	Original	Observer 1			Observer 2			Observer 3		
			NC	C	Kappa stats	NC	C	Kappa stats	NC	C	Kappa stats
Emax	Low	NC	1	0	κ= 0.200p= 0.505	1	0	κ= 0.545p= 0.171	1	0	κ= 0.500p= 0.248
			100.0%	.0%		100.0%	.0%		100.0%	.0%	
		C	2	1		1	3		1	2	
			66.7%	33.3%		25.0%	75.0%		33.3%	66.7%	
	Standard	NC	1	0	κ= 0.500p= 0.248	-	1	NA		1	NA
			100.0%	.0%			100.0%			100.0%	
		C	1	2		-	4			3	
			33.3%	66.7%			100.0%			100.0%	
	Low + MAR	NC	0	1	κ= -0.429p= 0.171	0	1	κ= -0.364p= 0.361	0	1	κ= -0.364p= 0.361
			.0%	100.0%		.0%	100.0%		.0%	100.0%	
		C	3	1		2	2		2	2	
			75.0%	25.0%		50.0%	50.0%		50.0%	50.0%	
	Standard + MAR	NC	0	1	κ= -0.500p= 0.386	0	1	κ= -0.429p= 0.171		1	NA
			.0%	100.0%		.0%	100.0%			100.0%	
		C	1	1		3	1			2	
			50.0%	50.0%		75.0%	25.0%			100.0%	
3Y	Low	NC	0	1	κ= -0.250p= 0.576	0	1	κ= -0.250p= 0.576	0	1	κ= -0.333p= 0.505
			.0%	100.0%		.0%	100.0%		.0%	100.0%	
		C	1	3		1	3		1	2	
			25.0%	75.0%		25.0%	75.0%		33.3%	66.7%	
	Standard	NC	1	0	κ= 0.286p= 0.361	0	1	κ= -0.364p= 0.361	0	1	κ= -0.500p= 0.386
			100.0%	.0%		.0%	100.0%		.0%	100.0%	
		C	2	2		2	2		1	1	
			50.0%	50.0%		50.0%	50.0%		50.0%	50.0%	
	Low + MAR	NC	0	1	κ= -0.333p= 0.505	0	1	κ= -0.250p= 0.576	0	1	κ= -0.333p= 0.505
			.0%	100.0%		.0%	100.0%		.0%	100.0%	
		C	1	2		1	3		1	2	
			33.3%	66.7%		25.0%	75.0%		33.3%	66.7%	
	Standard + MAR	NC	1	0	κ= 0.500p= 0.248	-	-	NA	0	1	κ= -0.333p= 0.505
			100.0%	.0%					.0%	100.0%	
		C	1	2		1	3		1	2	
			33.3%	66.7%		25.0%	75.0%		33.3%	66.7%	
5Y	Low	NC	0	1	κ= -0.333p= 0.505	0	1	κ= -0.250p= 0.576	0	1	κ= -0.333p= 0.505
			.0%	100.0%		.0%	100.0%		.0%	100.0%	
		C	1	2		1	3		1	2	
			33.3%	66.7%		25.0%	75.0%		33.3%	66.7%	
	Standard	NC	0	1	κ= -0.471p= 0.025*	0	1	κ= -0.800p= 0.083	0	1	κ= -1.000p= 0.157
			.0%	100.0%		.0%	100.0%		.0%	100.0%	
		C	4	0		2	0		1	0	
			100.0%	.0%		100.0%	.0%		100.0%	.0%	
	Low + MAR	NC	1	0	κ= 1.000p= 0.046*	-	-	NA	-	-	NA
			100.0%	.0%							
		C	0	3		-	4		-	4	
			.0%	100.0%			100.0%			100.0%	
	Standard + MAR	NC	1	0	κ= 0.286p= 0.361	-	-	NA	-	-	NA
			100.0%	.0%							
		C	2	2		2	2		1	2	
			50.0%	50.0%		50.0%	50.0%		33.3%	66.7%	

5

**Table 6:** PreXion® scanner with different materials and different settings for the mesial and distal positions. Different results were noted on the PreXion® scanner. The 3Y ranked 1st using both the Low and Standard setting showing statistically significant results with a positive association for Observers 2 and 3 (K 1.000, p 0.025 and K 1.000, 0.083 respectively) by the Low setting, and statistically significant results with positive association for Observers 1 and 3 (K 1.000, p 0.083 and K 1.000, p 0.046 respectively) by the Standard setting.

	Material	Setting	Original	Observer 1			Observer 2			Observer 3		
				NC	C	Kappa stats	NC	C	Kappa stats	NC	C	Kappa stats
Mesial	Emax	Low	NC	1	1	κ= -0.154p= 0.709	1	1	κ= 0.500p= 0.248	1	1	κ= 0.000p= 1.000
				50.0%	50.0%		50.0%	50.0%		50.0%	50.0%	
			C	2	1		0	2		1	1	
				66.7%	33.3%		.0%	100.0%		50.0%	50.0%	
		Standard	NC	2	-	NA	1	1	κ= -0.429p= 0.171	1	-	NA
				100.0%			50.0%	50.0%		100.0%		
			C	3	-		3	0		3	-	
				100.0%			100.0%	.0%		100.0%		
	3Y	Low	NC	2	0	κ= 0.615p= 0.136	2	0	κ= 1.000p= 0.025*	1	0	κ= 1.000p= 0.083
				100.0%	.0%		100.0%	.0%		100.0%	.0%	
			C	1	2		0	3		0	2	
				33.3%	66.7%		.0%	100.0%		.0%	100.0%	
		Standard	NC	1	0	κ= 0.500p= 0.248	1	1	κ= 0.545p= 0.171	1	0	κ= 1.000p= 0.083
				100.0%	.0%		50.0%	50.0%		100.0%	.0%	
			C	1	2		0	3		0	2	
				33.3%	66.7%		.0%	100.0%		.0%	100.0%	
	5Y	Low	NC	1	1	κ= -0.429p= 0.171	1	1	κ= -0.500p= 0.248	0	1	κ= -0.600p= 0.046
				50.0%	50.0%		50.0%	50.0%		.0%	100.0%	
			C	3	0		2	0		3	0	
				100.0%	.0%		100.0%	.0%		100.0%	.0%	
		Standard	NC	2	-	NA	2	0	κ= 0.286p= 0.361	2	-	NA
				100.0%			100.0%	.0%		100.0%		
			C	3	-		2	1		1	-	
				100.0%			66.7%	33.3%		100.0%		
Distal	Emax	Low	NC	0	1	κ= -0.250p= 0.576	-	1	NA	0	1	κ= -0.250p= 0.576
				.0%	100.0%			100.0%		.0%	100.0%	
			C	1	3		-	4		1	3	
				25.0%	75.0%			100.0%		25.0%	75.0%	
		Standard	NC	1	0	κ= 0.286p= 0.361	1	0	κ= 0.545p= 0.171	1	0	κ= 0.500p= 0.248
				100.0%	.0%		100.0%	.0%		100.0%	.0%	
			C	2	2		1	3		1	2	
				50.0%	50.0%		25.0%	75.0%		33.3%	66.7%	
	3Y	Low	NC	1	-	NA	1	0	κ= 0.286p= 0.361	1	-	NA
				100.0%			100.0%	.0%		100.0%		
			C	3	-		2	2		2	-	
				100.0%			50.0%	50.0%		100.0%		
		Standard	NC	1	0	κ= 1.000p= 0.083	1	0	κ = 0.545p = 0.171	1	0	κ= 1.000p= 0.046*
				100.0%	.0%		100.0%	.0%		100.0%	.0%	
			C	0	2		1	3		0	3	
				.0%	100.0%		25.0%	75.0%		.0%	100.0%	
	5Y	Low	NC	0	1	κ= -0.429p= 0.171	0	1	κ= -0.429p= 0.171	0	1	κ= -0.429p= 0.171
				.0%	100.0%		.0%	100.0%		.0%	100.0%	
			C	3	1		3	1		3	1	
				75.0%	25.0%		75.0%	25.0%		75.0%	25.0%	
		Standard	NC	0	1	κ= -0.471p= 0.025*	0	1	κ= -0.429p= 0.171	0	1	κ= -0.471p= 0.025*
				.0%	100.0%		.0%	100.0%		.0%	100.0%	
			C	4	0		3	1		4	0	
				100.0%	.0%		75.0%	25.0%		100.0%	.0%	

6

**Table 7:** Intra-observer reliability with statistically significant results. all p values were highly statistically significant <0.001 with Observer 1 having the highest ICC and the narrowest confidence interval suggesting excellent reliability, Observer 2 also demonstrated strong agreement but with a slightly wider confidence interval hinting to the presence of some variations, and Observer 3 had the lowest ICC and much wider confidence interval indicating a relatively lower but still good reliability.

Observer		Intraclass Correlation Coefficient (ICC)	95% CI	P Value
Observer 1	Mesial	0.979	0.956 – 0.990	<0.001**
	Distal	0.960	0.916 – 0.981	<0.001**
Observer 2	Mesial	0.914	0.821 – 0.959	<0.001**
	Distal	0.935	0.864 – 0.969	<0.001**
Observer 3	Mesial	0.883	0.758 – 0.944	<0.001**
	Distal	0.948	0.892 – 0.975	<0.001**

7

## Data Availability

The datasets used and/or analyzed during the current study are available from the corresponding author.
